# Zebrafish cyclin Dx is required for development of motor neuron progenitors, and its expression is regulated by hypoxia-inducible factor 2α

**DOI:** 10.1038/srep28297

**Published:** 2016-06-21

**Authors:** Huang-Wei Lien, Rey-Yue Yuan, Chih-Ming Chou, Yi-Chung Chen, Chin-Chun Hung, Chin-Hwa Hu, Sheng-Ping L. Hwang, Pung-Pung Hwang, Chia-Ning Shen, Chih-Lung Chen, Chia-Hsiung Cheng, Chang-Jen Huang

**Affiliations:** 1 Institute of Cellular and Organismic Biology, Academia Sinica, Taipei 115, Taiwan; 2 Department of Neurology, School of Medicine, College of Medicine, Taipei Medical University, Taipei 115, Taiwan; 3 Department of Biochemistry and Molecular Cell Biology, School of Medicine, College of Medicine, Taipei Medical University, Taipei 115, Taiwan; 4 Institute of Biological Chemistry, Academia Sinica, Taipei 115, Taiwan; 5 Department of Bioscience and Biotechnology, National Taiwan Ocean University, Keelung 20224, Taiwan; 6 Genomics Research Center, Academia Sinica, Taipei 115, Taiwan

## Abstract

Cyclins play a central role in cell-cycle regulation; in mammals, the D family of cyclins consists of cyclin D1, D2, and D3. In *Xenopus*, only homologs of cyclins D1 and D2 have been reported, while a novel cyclin, cyclin Dx (ccndx), was found to be required for the maintenance of motor neuron progenitors during embryogenesis. It remains unknown whether zebrafish possess cyclin D3 or cyclin Dx. In this study, we identified a zebrafish *ccndx* gene encoding a protein which can form a complex with Cdk4. Through whole-mount *in situ* hybridization, we observed that *zccndx* mRNA is expressed in the motor neurons of hindbrain and spinal cord during development. Analysis of a 4-kb promoter sequence of the *zccndx* gene revealed the presence of HRE sites, which can be regulated by HIF2α. Morpholino knockdown of zebrafish Hif2α and cyclin Dx resulted in the abolishment of *isl1* and *oligo2* expression in the precursors of motor neurons, and also disrupted axon growth. Overexpression of cyclin Dx mRNA in Hif2α morphants partially rescued *zccndx* expression. Taken together, our data indicate that zebrafish cyclin Dx plays a role in maintaining the precursors of motor neurons.

Cyclins and cyclin-dependent kinases (Cdks) were first identified in an effort to understand cell cycle control in different organisms, such as yeast, frogs, and mammals^1^,^2^,^3^,^4^,^5^,^6^. All cyclins have a conserved cyclin box domain that can bind to a similar region on Cdk^7^. In mammalian cells, cyclin D-Cdk4/6 is the first kinase to be activated by mitogenic signals that allow cells to exit G0 phase and enter G1 phase; this is followed by sequential activation of cyclin E-Cdk2 at the G1/S phase boundary, cyclin A-Cdk2 during S phase, cyclin A-Cdk1 during G2 phase, and finally, cyclin B-Cdk1 during mitosis^8^,^9^. Cyclins D1 and E are under mitogenic control, and are frequently deregulated during oncogenesis^8^.

Besides cell cycle control, cyclins, such as A1, A2, B1, B2, B3, D2, and D3, also play crucial roles in the control of mitotic and meiotic divisions during mammalian spermatogenesis^10^. In addition, cyclin E plays important roles in cell fate determination in the central nervous system^11^, while cyclin D1 can act as a transcriptional cofactor in a CDK-independent manner^12^.

In mammals, the cyclin D family consists of three members, cyclin D1, D2, and D3, which are also called G1-phase regulators^13^,^14^,^15^,^16^. In *Xenopus*, the cyclin D1 and cyclin D2 homologs have been cloned and characterized. At stage 33/34, cyclin D1 can be detected in eye, brain, branchial arches, neural tube, and tailbud, while cyclin D2 is also localized to the head region, including the brain, otic vesicle, nasal pits, and branchial arches^17^,^18^,^19^,^20^. Overexpression of cyclin D2 induces cell cycle arrest during early development^18^. However, a homolog of the cyclin D3 gene has not been identified in *Xenopus*. Instead, a novel *Xenopus* cyclin, cyclin Dx (*ccndx*), was recently identified, and found to be required for the maintenance of motor neuron progenitors (pMNs) during embryogenesis^21^.

In zebrafish, only cyclin D1 has been reported; this cyclin is expressed in G1 phase during development^22^, and is required for early eye development^23^. Down-regulation of cyclin D1 by morpholino (MO) injection was reported to cause microphthalmia and microcephaly^24^. Other cyclin D family members in zebrafish have not previously been reported. In this study, we identified and characterized a cyclin Dx homolog from zebrafish. The developmental and tissue-specific expression patterns of this *zccndx* mRNA were determined by RT-PCR and whole-mount *in situ* hybridization analysis. The promoter region of the zebrafish *zccndx* gene was also characterized, suggesting that it is regulated by Hif2α. Morpholino knockdown of *zccndx* or *Hif2α* expression demonstrated the importance of these genes in maintaining the precursors of motor neurons.

Initiation of anti-neural factor expression expands the pool of neural progenitors; subsequent cell cycle exit and differentiation results in the development of primary motor neurons. Expression of two genes, *Islet1* (*Isl1*) and *oligo2* (*olig2*), is required to maintain a pro-neurogenic state. Isl1 is required for motor neuron generation in vertebrates^25^, loss of zebrafish isl1 leads to the loss of primary motor neurons^26^. On the other hand, olig2 is required for primary motor neuron differentiation of neural precursor cells^27^; phosphorylation of olig2 maintains cells in a pro-neurogenic state^28^. Both genes are essential for primary motor neuron development. In this study, we identified that the zebrafish *ccndx* gene acts as a cell-cycle regulator to maintain the pool of motor neuron progenitors, as previously reported for *Xenopus*^21^. Furthermore, knockdown of *zccndx* resulted in loss of differentiated motor neurons, and ultimately, the disruption of axon growth.

## Experimental Procedures

### Fish

The Tg (isl:GFP) transgenic line^29^ was obtained from Zebrafish Core Facility at NHRI (National Health Research Institutes). Zebrafish (*Danio rerio*) were maintained at 28 °C on a 14 h-light/10 h-dark cycle. Embryos were incubated at 28 °C, and different developmental stages were determined according to the description provided in the *Zebrafish Book*^30^. All animal procedures were approved by Academia Sinica Institutional Animal Care and Utilization Committee (ASIACUC) (protocol #10-12-114). The methods were carried out in accordance with the approved guidelines.

### Cell culture

Monkey kidney fibroblast COS-1 cells and human embryonic kidney HEK 293T cells were cultured in high-glucose Dulbecco’s modified Eagle’s medium (DMEM), supplemented with 10% fetal bovine serum (FBS; HyClone, UT, USA) in a humidified atmosphere of 5% CO_2_ at 37  °C.

### Cloning of the full-length cDNAs encoding zebrafish *cyclin dx* and *cyclin d1*

The cDNA encoding the complete open reading frame (ORF) of zebrafish *ccndx* (*cyclin dx*), *ccnd1* (*cyclind1*), and *smo* were obtained by PCR amplification using gene-specific primers, which were designed based on the NCBI’s zebrafish EST database. DNA fragments containing full-length cDNA were obtained by PCR amplification using forward primer (ccndx-F1: 5′-GGA CAG ACT TGA GTG TTG GAG CAG-3′; ccnd1-F1: 5′-AAC ACT GAA GAT ATG GAG CAC CAG-3′) and reverse primer (ccndx-R1: 5′-TAT ACG AGT GAT GAA TGA ACC GCG-3′; ccnd1-R1: 5′-ACA CAC GAG ATG AAT AGC GAT CCC-3′). The PCR products were subcloned into the pGEM-T Easy vector (Promega, WI, USA), and the resulting clones were subjected to sequence analysis.

### Luciferase assay

The 2,432bp upstream region of the zebrafish *cyclin dx* gene was amplified from BAC clone, CH211-152F23 (RZPD, Berlin, Germany), using forward primer (F1: 5′- GGT ATC CCT GGA CCT TGT ATA CGC AGTGGT-3′) and reverse primer (R1: 5′- CAA TGT GTC CTG TTG CAC GTG TGT CTT GTG-3′). The amplified fragments were then inserted into pGEM-T Easy cloning vector (Promega, WI) for sequencing and subsequently subcloned into the *Kpn*I/*Nco*I sites of the pGL3 vector to generate pGL3-ccndx-2.5k-pro plasmid. Reduced promoter region of pGL3-ccndx-2.5 kb to 1,096bp by restriction enzyme digestion in *Hind*III and *Kpn*I sites was generated pGL3-1 b-pro plasmid. The pGL3-0.7 -pro plasmid was generated by reduced promoter region to 654bp using *Sac*I and *Kpn*I sites. Constructs containing mutated HRE elements were generated using the following primers: pGL3-ccnx-0.7 -pro-HRE1-Mut – 0.7KPROF (5′-CAC GGT ACC CAC AGT TTT CGA GGG ATA C-3′), HRE1MF (5′-CAC GGT ACC CAC AGT TTT CGA GGG ATA C-3′) and HRE1MR (5′-AAT CTC GAG CAA ATC TGT CCT GTT GAT ATC G-3′); 0.7KPROF (5′-CAC GGT ACC CAC AGT TTT CGA GGG ATA C-3′), 0.7KPROR (5′-AAT CTC GAG CAA TGT GTC CTG TTG CAC GTG-3’), pGL3-ccnx-0.7 -pro-HRE2-Mut – HRE2MF (5′-CAA AAT ATC TTC TTT TTG ATA TCA ACA TTT-3′) and HRE2MR (5′-AAA TGT TGA TAT CAA AAA GAA GAT ATT TTG-3′). The 5 × 10^5^ COS-1 cells were placed in each of 12-well plate overnight before transfection. Cells was transfected with 0.5 g of pGL3-2.5k-pro, pGL3-1k-pro, pGL3-0.7k-pro, pGL3-0.7k-pro-HRE1-Mut, pGL3-0.7k-pro-HRE2-Mut plasmid along or co-transfected with 1 g of pcDNA3-Hif2α-HA and pcDNA3-Arnt1α-HA using the Lipofectamine Plus reagent (Invitrogen), according to the manufacturer′s instructions. Luciferase activity were measured at 48 h post-transfection using the ONE-GLO Luciferase Assay Reagent (Promega, WI, USA).

### Electrophoretic mobility shift assay

After COS-1 cells were transfected with pcDNA3-HA or pcDNA3-Hif2α-HA and pcDNA3-Arnt1α-HA plasmids for 48 h, nuclear extracts were prepared by minipreparation method as described previously^31^ The nuclear extracts were storage at −70 °C until use. The HRE1 and HRE2 sites of the *ccndx* promoter were used as probes for EMSA experiment. HRE1 (5′-CAC AAG ACA CAC GTG CAA CAG GAC ACA TTG-3′) and HRE2 (5′- CAA AAT ATC TTC TTT TTG CGT GCA ACA TTT-3′) core sequence and corresponding mutant oligonucleotides were synthesized with or without labeled at the 5′-end with biotin (Genomics, Taipei, Taiwan). For each EMSA experiment, 20 g of nuclear extracts were incubated with HRE1 or HRE2 probe and Binding Reagents (Thermo, IL, USA) at room temperature for 30 minutes. For competitive experiments, 50X normal or mutant probes were added. The DNA-protein complexes were separated on a non-denaturing 10% polyacrylamide gel. The gel was transferred to a nylon membrane (Pall, NY, USA), followed by UV cross-linking and detection with a LightShift Chemiluminescent EMSA Kit (Thermo, IL, USA).

### Plasmid construction

The expression vectors, pcDNA3-*ccndx*-*myc* and pcDNA3-*ccnd1*-*myc*, were generated by inserting full-length *cyclin dx* and *cyclin d1* cDNA between *Bam*HI and *Eco*RI sites of pcDNA3-*myc*-*HisA* plasmid, respectively. The pcDNA3-*cdk4*-*HA* plasmid was constructed by inserting the full-length *cdk4* cDNA between the *Bam*HI and *Eco*RI sites of the pcDNA3-*HA*-*YUN* plasmid.

### Immunoblotting and co-immunoprecipitation analysis

Total cell lysate was collected with IP-lysis buffer (150 mM NaCl, 20 mM NaCl, 20 mM HEPES, pH 7.2, 10 mM NaF, 1 mM EDTA, 1% NP-40, 1 mM Na_3_V0_4_, 1 mM PMSF, 1  DTT, and proteinase inhibitor cocktail) at 72 h, and subjected to immunoblotting with anti-myc (1:3000, Santa Cruz, CA, USA) and anti-HA (1:1000, Santa Cruz, CA, USA) monoclonal antibody. Co-immunoprecipitation was performed with 25 μl Protein A/G PLUS-Agarose (Santa Cruz, CA, USA) and anti-myc monoclonal antibody (1 μg) to pull-down the complex, which was then immunoblotted with anti-HA monoclonal antibody.

### Capped mRNA synthesis

Full-length cDNA fragments of zebrafish ccndx (JX099349.1), mouse ccnd1 (NM_007631.2), ccnd2 (NM_009829.3), and ccnd3 (NM_001081635.1) were amplified and inserted into the pCS2 + vector. Each of the resulting pCS2 + plasmids was linearized with NotI. Capped mRNA was transcribed from 1 μg linearized plasmid using the mMESSAGE mMACHINE SP6 Kit (Ambion, Foster City, CA, USA).

### Morpholino knockdown of *hif2α* and *ccndx*

The antisense morpholino oligonucleotides (MO) used to knockdown *hif2α* were designed according to a previous report^32^. The sequences of the *ccndx* MOs were as follows: ccndx ATG MO- 5′ ATC ACT GCT CCA ACA CTC AAG TCT G 3′; ccndx splicing MO- 5′- TGG TTT ATG ATA TCT AAA CAC TAC C 3′. The control MO sequence was as follows: 5′- CCT CTT ACC TCA GTT ACA ATT TAT A-3′. All antisense MOs were synthesized by Gene Tools (Philomath, OR, USA). Each MO was dissolved in 1x Danieau solution (5 mM HEPES, pH 7.6, 58 mM NaCl, 0.7 mM KCl, 0.4 mM MgSO4, and 0.6 mM (NO3)2) containing 0.5% phenol red to a concentration of 0.3 mM.

### Injection of morpholinos and capped mRNA

MOs and capped mRNA were injected into wild-type zebrafish embryos using a microinjection system consisting of a SZX9 stereomicroscope (Olympus, Tokyo, Japan) and an IM300 Microinjector (Narishige, Tokyo, Japan). The capped mRNA was injected into embryos at the 1-cell stage, while MOs were injected at the 2- to 4-cell stage of wild-type zebrafish embryos. The MO-mediated effects were evaluated at 24 h after injection.

### Whole-mount immunostaining

Whole-mount immunostaining was performed following standard protocols as previously described^33^ with some modifications. The antibodies used were as follows: mouse monoclonal anti-synaptotagmin antibody against znp1^34^ (Developmental Studies Hybridoma Bank (DSHB), Iowa City, IA) and Cy2-conjugated anti-mouse IgG (Jackson ImmunoResearch Laboratories, Inc., West Grove, PA).

### Whole-mount *in situ* hybridization

To synthesize digoxigenin-labeled (Roche, Penzberg, Germany) antisense RNA probes, we linearized pGEM-T easy-*ccndx* with *Pst*I and transcribed it with T7 RNA polymerase. The probes for motor neuron progenitors and interneurons (*isl1*, *olig2*, *nkx6.1*, and *vsx1*) have been described previously^27^,^35^,^36^,^37^; these probes were linearized with *Nco*I and transcribed with SP6 RNA polymerase. To generate fluorescein-labeled (Roche, Penzberg, Germany) antisense probes, we linearized pcDNA3-CMV-GFP with *Hind*III and transcribed it with SP6 RNA polymerase. Whole-mount *in situ* hybridization was performed as previously described^38^,^39^. For double fluorescence *in situ* hybridization, the digoxigenin-labeled probes were detected using anti-digoxigenin POD (Roche, Penzberg, Germany) (1:500 dilution), and signals were amplified using a tyramide-FITC kit (Perkin Elmer, Boston, MA, USA). After the first staining step, the tyramide working solution was washed away and the embryos were incubated for 30 minutes with 1% H_2_O_2_, to inactivate the peroxidase activity of the first antibody. The embryos were then blocked for 1 hour, and the fluorescein-labeled probes were detected with anti-fluorescein POD (Roche, Penzberg, Germany) (1:500 dilution); the signals were subsequently amplified using a tyramide-Cy2 kit (Perkin Elmer, Boston, MA, USA).

### Proliferating cell labeling and quantification

Proliferating cells were labeled with rabbit anti-pH3 (Cell signaling Technology) and BrdU (Sigma) as previously described^40^,^41^; and goat anti-mouse-IgG conjugated to Cy2 and Cy3 (Invitrogen, USA) were utilized in our study. The fluorescent images were acquired on a Zeiss LSM 710 laser confocal microscope. The labeled proliferating cells within the image of the developing brain were counted through ImageJ analysis. All the statistical analysis was performed by SPSS. Significance was evaluated by one-way ANOVA while considered differences at P < 0.05.

### High resolution confocal imaging

Zebrafish embryos were fixed with 4% paraformaldehyde in PBS at 4 °C for 24 , and then washed 3 times in PBS (5 min/wash). Embryos were cleared as described previously^42^; embryos were incubated in FocusClear in a small chamber with a spacer ring for 5 min, and then mounted with MountClear at room temperature for 30 min. Images were captured with a Zeiss LSM 710 confocal microscope (Carl Zeiss, Jena).

## Results

### Cloning and characterization of zebrafish cyclin Dx

Mammals possess three members of the cyclin D family: cyclin D1, D2, and D3^13^,^14^,^43^,^44^. In *Xenopus*, cyclin D1 and D2 have been identified^17^,^45^, but not cyclin D3. Instead, a novel cyclin D, cyclin Dx, was recently identified in *Xenopus*^21^. From an evolutionary perspective, zebrafish may be expected to possess cyclin D family members similar to those of *Xenopus*. However, only cyclin D1 has been identified in zebrafish^24^,^46^. To search for zebrafish cDNAs related to *Xenopus cyclin Dx* and *cyclin D2*, we used the coding regions of *Xenopus cyclin Dx* (accession no. AAI58908) and *cyclin D2* (P53782) to BLAST search GenBank for related expression sequence tag (EST) sequences. Sequence assembly and 5′-RACE enabled the identification of one zebrafish *cyclin Dx* and two cyclin D2-related cDNAs, designated *zccnDx*, *zccnD2A*, and *zccnD2B*, respectively; the assembled sequences were deposited in GenBank with the following accession numbers: FJ648072, *zccnDx*; FJ648073, *zccnD2A*; and FJ648074, *zccnD2B*. The full-length coding regions of each cDNA were amplified by PCR using gene-specific primers, and were confirmed by sequence analysis.

**Figure 1:**
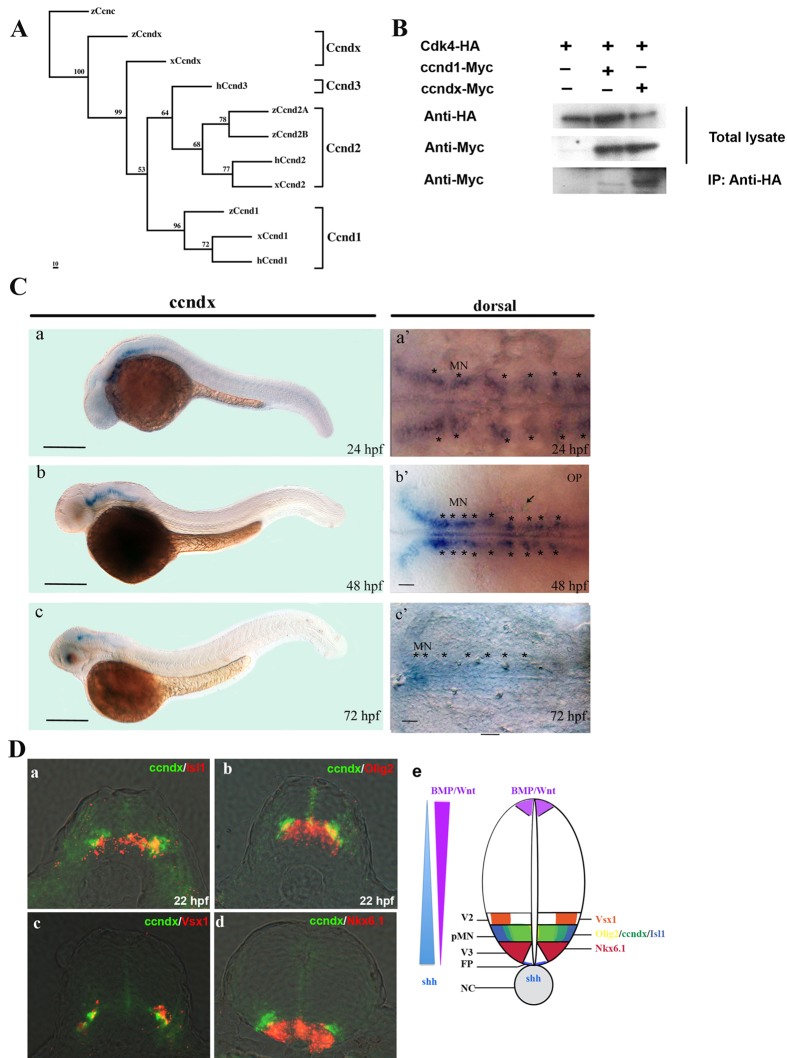
Zebrafish cyclin Dx is a member of the cyclin D family, and *zccndx* is expressed during early development. (**A**) Phylogenetic tree analysis of zccndx with other cyclin D proteins. Four groups of the cyclin D family, ccnD1, D2, D3, and Dx, exhibited perfect separation. The zccndx protein was grouped with *Xenous* ccndx. Analysis was performed using the parsimony method with 1000 bootstraps. (**B**) The zccndx protein binds to Cdk4. COS-1 cells were cotransfected with the indicated constructs, and cell lysates were co-immunoprecipitated (IP) with anti-HA monoclonal antibody; the anti-Myc monoclonal Ab was used for detection. The immunoblot was probed using the indicated antibodies. (**C**) At 24 hpf, *zccndx* mRNA was observed to be expressed in the motor neurons of hindbrain and spinal cord (a and a′). At 48 hpf, *zccndx* mRNA was also expressed in the hindbrain, but not in the spinal cord (b and b′). At 72 hpf, *zccndx* mRNA was weakly expressed in the hindbrain, with no signal in the spinal cord (c and c′). Dorsal views of *zccndx* mRNA signal in the hindbrain are shown (a′–c′). Scale bar, 100 mm. (**D**) Sections were made in the hindbrain at 22 hpf. The *zccndx* signal is shown in green and the *isl1* (panel a), *olig2* (panel b), and *vsx1* (panel c), and *nkx6.1* (panel d) mRNA signals are shown in red. The cartoon (panel e) shows the location of *zccndx* (green), *isl1* (purple), *olig2* (orange), *vsx1* (blue), and *nkx6.1* (red) signals. The Shh (blue) signaling gradient has been created from the floor plate to diffuse along the ventral tube while BMP/Wnt (purple) gradient shows opposite direction. The interaction of the dorsalizing and ventralizing signals defines the proper position of motoneurons. Scale bar, 100 mm.

An alignment of the deduced amino acid sequences of zebrafish, *Xenopus* and human cyclin D-related proteins is shown in Supplemental Fig. 1. The zebrafish cyclin Dx protein contains 297 amino acid residues, and displayed low identity to all examined cyclin D-related proteins (31% to 35% identity; 50% to 53% similarity). The *zccnD2A* and *zccnD2B* cDNA sequences are predicted to encode proteins of 298 and 296 amino acid residues, respectively. The zebrafish cyclin D2A protein exhibits higher identity to human and *Xenopus* cyclin D2 (79% and 80% identity, respectively). On the other hand, zebrafish cyclin D2B protein displays 68% identity to human and *Xenopus* cyclin D2. Interestingly, zebrafish cyclin D2B is also homologous to cyclin D2A with 72% identity, suggesting that they may be derived from a gene duplication event. The cyclin box domain of cyclin Dx was conserved with that of other cyclin D family members. Phylogenetic tree analysis, as shown in Fig. 1A, indicated that zebrafish cyclin Dx is grouped with *Xenopus* cyclin Dx, but is separate from other cyclin D family members.

*Xenopus* cyclin Dx has been reported to bind to Cyclin-dependent kinase 4 (Cdk4) in HEK 293T cells^21^. Based on this report and the above finding, we hypothesized that zebrafish cyclin Dx may be a member of the cyclin D family, and bind to Cdk4. Indeed, co-immunoprecipitation experiments revealed that zebrafish cyclin Dx binds to Cdk4 in COS-1 cells (Fig. 1B).

### Spatial and temporal distribution of zebrafish cyclin Dx mRNA during development

Whole-mount *in situ* hybridization using the 3′-UTR region of *zccndx* gene as probe was performed to detect the expression pattern of *zccndx* mRNA during development. At 24 hpf, *zccndx* mRNA was expressed on both sides of the midline in hindbrain and spinal cord (Fig. 1C, a and a′). At 48 hpf, *zccndx* mRNA continued to be expressed in the hindbrain, but the signal in the spinal cord became weak (Fig. 1C,b,b′). At 72 hpf, the expression of *zccndx* mRNA was very weak in the hindbrain, and no signal was detected in the spinal cord (Fig. 1C,c,c′). The expression pattern of *zccndx* mRNA is similar to that of *xccndx* mRNA, which has been reported to be expressed in the progenitors of motor neurons (pMNs)^21^. Markers for pMNs, such as *isl1*^35^ and *olig2*^27^, were subsequently used to determine co-localization of *zccndx* mRNA expression in the experiments described below.

### The zebrafish *cyclin Dx* gene is expressed in the motor neuron progenitor region

During the early development of zebrafish, motor neuron progenitors (pMNs) are distributed in the ventral neural tube between the V2 and V3 interneuron progenitors. The *isl1* and *olig*2 genes are significantly expressed in this neural tube layer, and were thus used as markers to identify pMNs^27^,^35^. The *vsx1* gene is expressed in the V2 interneuron progenitor layer in the neural tube, and was thus used as the interneuron marker^47^. Finally, the *nkx6.1* gene is expressed in V3 interneuron progenitors^48^.

We performed double *in situ* hybridization of zebrafish embryo sections to investigate whether the *zccndx* gene is expressed in the pMN layer. Different markers for the progenitors of motor neurons and interneurons in the ventral spinal cord, such as *isl1*, *olig*2, *vsx1*, *nkx6.1*, and endogenous *zccndx*, were double labeled with Cy3 and Cy5 at 22 hpf. The *zccndx* mRNA was observed to be expressed in the ventral spinal cord (Fig. 1D, panel a). The *isl1* mRNA signal in the pMN layer of the ventral spinal cord partially overlapped with the *zccndx* mRNA signal in the same layer (Fig. 1D, panel a). An *olig2* mRNA signal was also observed in the pMN layer of the ventral spinal cord, and this signal partially overlapped with the *zccndx* mRNA in the same layer (Fig. 1D, panel b). A *vsx1* signal was observed in the V2 layer of the ventral spinal cord; this signal was above the *zccndx* mRNA signals, and the two signals did not overlap (Fig. 1D, panel c). Another marker of the V3 layer of interneuron progenitors, *nkx6.1* mRNA, was observed to be expressed below the *zccndx* mRNA signal (Fig. 1D, panel d). Taken together, our data demonstrate that expression of *ccndx* mRNA is restricted to the pMN layer of ventral spinal cord, and partially overlaps with the expression of *olig2* and *isl1* mRNA (Fig. 1D, panel e).

### The *ccndx* gene is required for the maintenance of motor neuron progenitors

**Figure 2:**
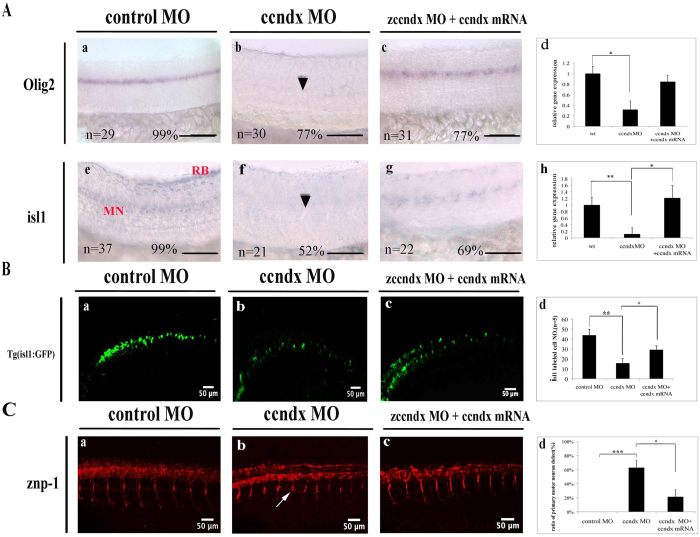
Morpholino (MO) knockdown of *zccndx* affects motor neuron development. (**A**) MO knockdown of *zccndx* reduced the *olig2* and *isl1* signals in motor neurons (panels a, b, e, and f); rescue experiments performed by co-injecting *ccndx* mRNA are shown in panels c and g. Panels d and h: graphs showing the qPCR analysis confirmed the results of morphants with signals lower than control MO-injected embryos at 24 hpf. **P < 0.05. Scale bar, 100 mm. (**B**) The *ccndx* morphant showed a significantly higher ratio of embryos with lower numbers of GFP-labeled primary motoneurons than the control MO-injected embryos (panels a and b) in the Tg:(isl1:GFP) transgenic line. The ccndx mRNA recue experiment is shown in panel c. Panel d: the Statistical charts represent the quantitative results of GFP-labeled primary motoneurons cells at 24 hpf. (**C**) Labeling of primary motor axons with Znp1 antibody revealed that CaP axon branches were shorter in *ccndx* morphants than in control morphants (panels a and b); the rescue experiment is shown in panel c. Panel d: graphs showing the percentages of morphants with primary motor defects as compared to control MO-injected embryos at 24 hpf in panels d and h. *P < 0.05.

To explore the functions of zebrafish cyclin Dx, we performed MO knockdown of the *ccndx* gene; this resulted in the loss of motor neurons. To determine the specificities of the MOs used, we created pCMV-GFP reporter plasmids. The 25-bp target sequence of the ATG MO was cloned upstream of the GFP open reading frame (ORF) in the pCMV-GFP reporter plasmid. We injected zebrafish embryos with a pCMV-GFP reporter plasmid containing the MO target sequence, with or without *ccndx* ATG-MO (Supplementary Fig. 2A, panels a and b). The GFP signal intensity was decreased in MO-injected embryos as compared to embryos uninjected with MO, confirming the specificity of *ccndx* MO; in addition, levels of *ccndx* were shown to be reduced by the MO (Supplementary Fig. 2A,B). While 81.1% and 68.3% of *ccndx* morphant embryos exhibited decreased expression of *olig2* and *isl1* signals in motor neurons as compared to this control, these percentages could be decreased to 15% and 37.6% by co-injection of *ccndx* mRNA, indicative of partial rescue (Fig. 2A, panels a–h). In addition, we analyzed the effects of the loss of *ccndx* on motoneurons by using the Tg:(isl1:GFP) transgenic line. GFP expression was observed in all the cranial motoneurons and primary motoneurons located in the ventral region of the spinal cord within control MO-injected embryos at 72 hpf (Fig. 2B, panel a). However, *ccndx* morphant embryos exhibited lower numbers of GFP-labeled primary motoneurons compared to control MO-injected embryos (Fig. 2B, panels b and d). This phenotype could be partially rescued by injection of *ccndx mRNA* (Fig. 2B, panels c and d). To determine whether *ccndx* is required for normal outgrowth by primary motor axons, we injected *ccndx* MO into fertilized embryos and assayed primary motor axons with antibody of znp1 after early development. We observed that 62.8% of *ccndx* morphants exhibited shorter CaP axon branches compared with control morphants (Fig. 2C, panel b). Again, injection of *ccndx* mRNA could partially rescue this phenotype (Fig. 2D, panels c and d). Thus, *zccndx* functions in maintaining the expression of motor neuron progenitors, which are specifically required for subsequent primary motor neuron formation during development in the ventral spinal cord, and is also necessary for the subsequent appropriate outgrowth of CaP axons.

**Figure 3:**
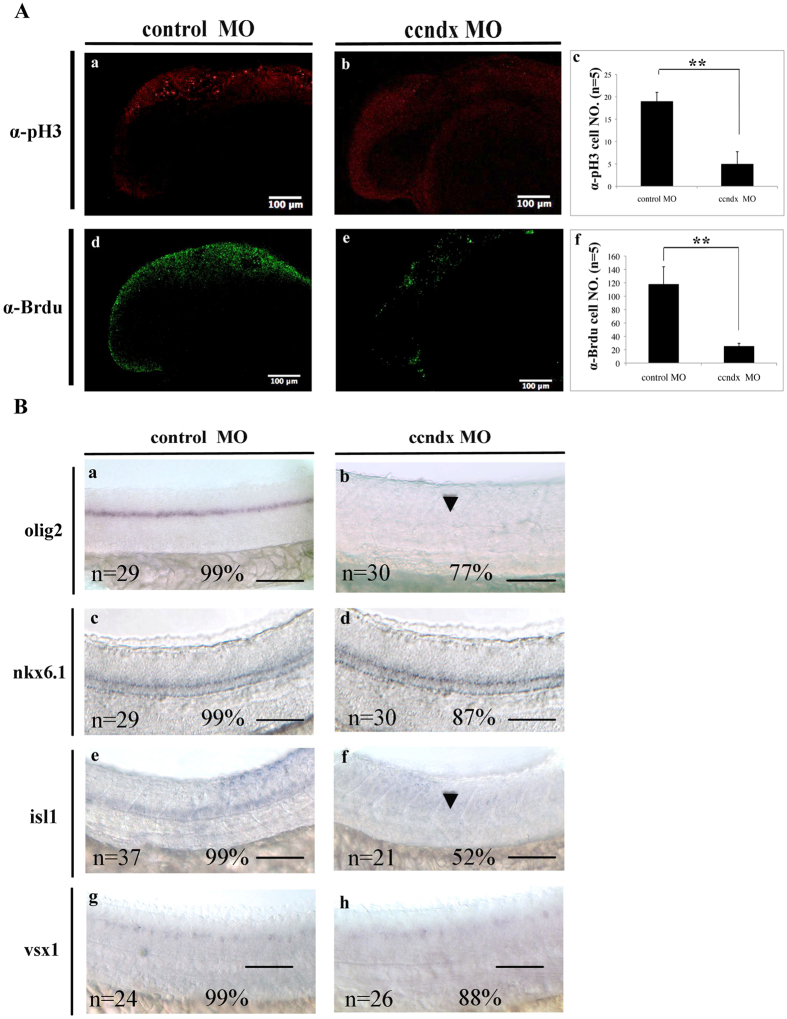
The role of *zccndx* in cell proliferation and regulation of the numbers of motor neuron progenitors (pMNs). (**A**) Confocal images showing lateral views of the heads of control-MO-injected (panel a) or ccndx-MO-injected (panel b) embryos stained with anti-pH3 (red in panels a and b) or anti-BrdU (green in d and e). Panels c and f: graphs showing the number of proliferating cells (pH3 or BrdU positive) at 24 hpf. The asterisks indicate significant differences between the control and experimental morphants. The n value is indicated. (**B**) Whole–mount *in situ* hybridization of motor neurons and interneuron markers in control-MO and *zccndx* ATG-MO-injected embryos. Probes against *olig2* (a and b), *nkx6.1*(c and d), *isl1* (e and f), and *vsx1* (g and h) were used for detection. control MO-injected embryos are shown in panels a, c, e and g while *ccndx* ATG-MO-injected embryos are shown in panels b, d, f and h. Motor neurons (MN) were lost (panels b, f, lost MNs indicated by arrowheads), while interneurons (IN) (panels d and h) were unaffected. Scale bar, 100 mm.

We then compared the cell proliferation statuses of *ccndx* mutants and *ccndx* morphants by utilizing anti-phosphohistone 3 (pH3) antibody staining and BrdU labeling. The cell proliferation assays showed that at 24 hpf, the pH3-positive cell numbers in the brain region of *ccndx* morphants were significantly reduced compared to control MO-injected embryos (Fig. 3A, panels a and b). Consistent with the findings of the anti-pH3 assay, the numbers of BrdU-positive cells in brain region in *ccndx* morphants were reduced compared to those in control MO-injected embryos at 24 hpf (panels d and e). These data indicate that *ccndx* modulates cell proliferation in the brain region. To further investigate the effect of *ccndx* on motor neuron precursors, we injected *ccndx* MO into the embryos of wild-type zebrafish at 2–4 cell stages. At 24 hpf, MO-injected embryos were collected and subjected to whole-mount *in situ* hybridization using different markers for each progenitor domain of motor neurons and interneurons in the ventral spinal cord, namely *isl1* (pMN), *olig2* (pMN), *vsx1* (V2), and *nkx6.1* (V3). In *ccndx* morphants, the *isl1* and *olig2* signals in motor neurons were lost (Fig. 3B, panels a, b, e, and f). In contrast, *ccndx* MO-injected embryos (panels c and g) displayed similar expression patterns of *nkx6.1* and *vsx1* mRNA to those observed in the control MO-injected embryos (panels c and d).

### Expression of the zebrafish *ccndx* gene is regulated by HIF2α through HRE sites

**Figure 4:**
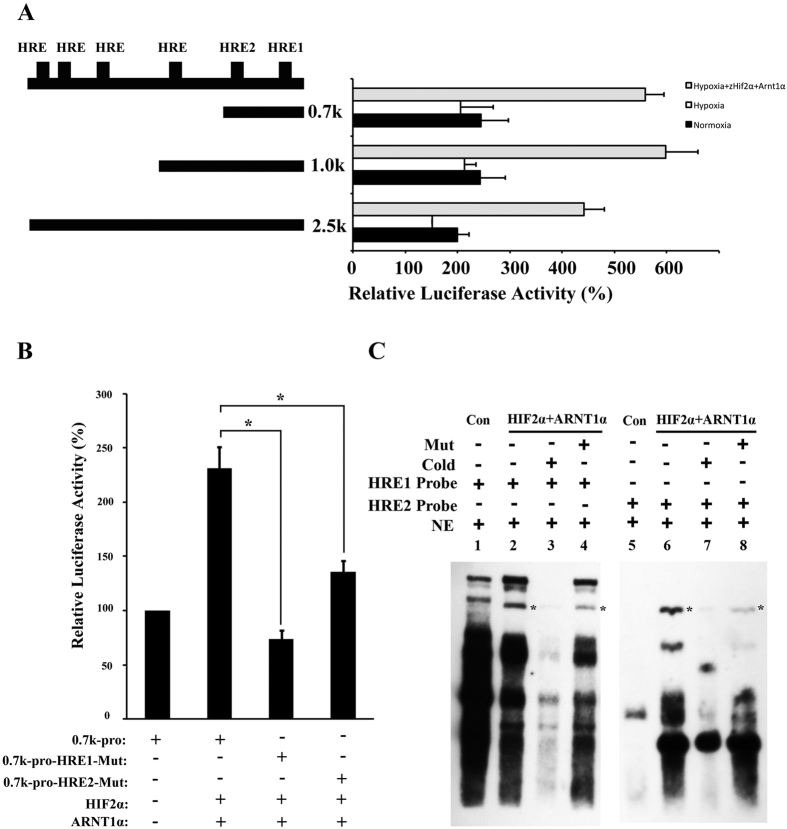
Expression of the *ccndx* gene is regulated by the HIF2α transcription factor. (**A**) The promoter region of the *ccndx* gene contains several HRE sites, as indicated in the schematic diagram (top left). The 2.5-kb, 1-kb, and 0.7-kb promoters are all activated by HIF2α and arnt1a transcription factors. (**B**) The HIF2α and ARNT1α proteins co-activate *ccndx* gene expression through two HRE sites in the 700bp promoter region. Data represent mean ± s.d. (n = 3). (**C**) The DNA binding ability of HIF2α and the ARNT1α complex were confirmed by EMSA. Nuclear extract (NE) from pcDNA3-HA (Con) or pcDNA3-Hif2α-HA and pcDNA3-Arnt1α-HA (HIF2α + ARNT1α) transfection were indicated. Two HRE oligonucleotides (as indicated) were used as probes and normal (Cold) or mutant (Mut) oligonucleotides were used for competition. The asterisks in lanes 2, 4, 6, and 8 indicate the specific binding complex.

In a previous report, HIF2α was found to regulate *birc5a*/*5b* gene expression by directly binding to Hypoxia-Response Elements (HREs) in their promoter region, thereby protecting neural progenitor cells^32^. We report here that *ccndx* is required for the maintenance of pMNs, and that knockdown of *ccndx* mRNA expression causes apoptosis of pMNs. Analysis of the upstream region of the *ccndx* gene revealed several HRE sites. Two HRE sites are located in the upstream region of 654 bp (0.7 ) of *ccndx* gene. Transactivation analysis using luciferase assays in COS-1 cells revealed significant activation of this 0.7  promoter by HIF2*α* and ARNT1*α* protein (Fig. 4A). Mutation of these two HREs reduced transactivation activity (Fig. 4B). These data suggest that HIF2*α* regulates *ccndx* gene expression through HRE sites. To determine whether zebrafish HIF2*α* specifically binds to the HRE sites in the *ccndx* promoter, we performed electrophoretic mobility shift assay (EMSA) with biotin-labeled oligonucleotides for each of the two HRE core sequences (ACGTG) (Fig. 4C). When HIF2*α* was expressed together with ARNT1*α* in COS-1 cells, a mobility complex was formed with each of the two wild-type HRE probes (lanes 2 and 6). This mobility complex was successfully out-competed by a 200-fold molar excess of the unlabeled HRE (lanes 3 and 7). On contrary, the mobility complex was unaffected by a 200-fold molar excess of unlabeled mutated HRE (lanes 4 and 8). These results thus demonstrate that HIF2*α* binds specifically to each of the two HRE sites.

### The HIF2α transcription factor regulates *ccndx* gene expression, which is required for motor neuron development

**Figure 5:**
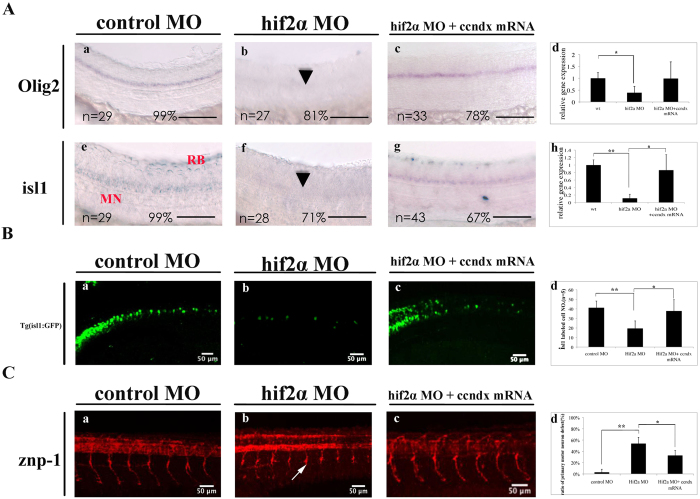
The HIF2α transcription factor helps regulate *ccndx* gene expression and subsequent motor neuron development. (**A**) MO knockdown of *hif2a* reduced the *olig2* and *isl1* signals in motor neurons (panels a, b, e, and f); co-injection of *ccndx* mRNA partially rescued this phenotype (panels c and g). Panels d and h: graphs showing the qPCR analysis confirmed the results of morphants with signals lower than control MO-injected embryos at 24 hpf. *P < 0.05. Scale bar, 100 mm. (**B**) A significantly higher proportion of hif2a morphant embryos had lower numbers of GFP-labeled primary motoneurons as compared to control MO-injected embryos (panels a and b) in the Tg:(isl1:GFP) transgenic line. The effect of rescue experiments with *ccndx* mRNA is shown in panel c. Quantitative data at 24 hpf are shown in panel d. (**C**) A significantly higher proportion of hif2a morphant embryos exhibited shorter CaP axons branches as compared to control MO-injected embryos (panels a and b). The effect of rescue experiments with *ccndx* mRNA is shown in panel c. Quantitative data at 24 hpf are shown in panel d. *P < 0.05.

To explore how HIF2*α* and cyclin Dx regulate the formation of motor neuron progenitors expressing *olig2* and *isl1*, we performed rescue experiments by injecting mRNA into MO knockdown morphants. The *olig2* and *isl1* signals were lost from the majority of HIF2α morphants (Fig. 5A, panels b and f); motor neuron progenitor cells (expressing *olig2* and *isl1*) were partially restored by co-injection of *zccndx* mRNA (Fig. 5A, panels c and g, d and h). Similar effects were observed in GFP-labeled cranial motoneurons and primary motoneurons cells of the Tg(Isl1:GFP) transgenic line; HIF2α morphants exhibited reduced GFP-labeled primary motoneurons cells number as compared to control MO-injected embryos (Fig. 5B, panels b and d), while the number of GFP-labeled motoneurons could be rescued by injection of *ccndx* mRNA (Fig. 5B, panels c and d). Rescue was also observed for Znp1 antibody-labeled primary motor axons during early development. We found that 53.8% of HIF2α morphants had shorter CaP axon branches than those of control morphants (Fig. 5C, panels b and d), and this could be partially reversed by injection of *ccndx* mRNA (Fig. 5D, panels c and d). Taken together, these results suggest that *ccndx* gene is regulated by HIF2α, which functions in maintaining the expression of *olig2* and *isl1*, and the subsequent formation of primary motor axons.

### Mouse cyclin D1, D2, and D3 cannot restore the expression of *isl1* and *olig2* in the motor neuron progenitor region in the absence of zebrafish cyclin Dx

**Figure 6:**
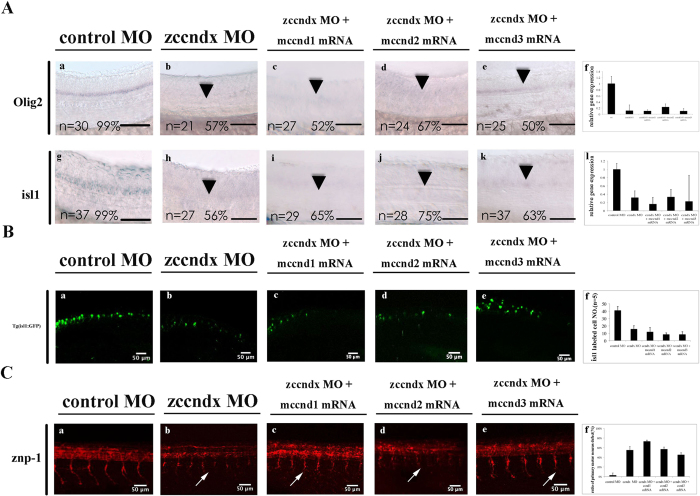
Mouse cyclin D1, D2, and D3 cannot compensate for the loss of zebrafish cyclin Dx. (**A**) MO knockdown of *ccndx* reduced the *isl1* and *olig2* signals in motor neurons (panels a, b, g, and h); co-injection of mouse *ccnd1*, *ccnd2*, and *ccnd3* mRNA had no effect, as shown in panels c to e and i to k. Panels r and l: graphs showing the qPCR analysis confirmed the results of morphants with signals lower than control MO-injected embryos at 24 hpf. *P < 0.05. Scale bar, 100 mm. (**B**) A significant number of *ccndx* morphant embryos contained fewer GFP-labeled primary motoneurons as compared to *ccndx* morphants (panels a and b) in the Tg:(isl1:GFP) transgenic line, and co-injection of mouse *ccnd1*, *ccnd2*, or *ccnd3* mRNA was unable to rescue this defect (panels c, d, and e). Panel f: graph showing the quantitative results of GFP-labeled primary motoneurons cells at 24 hpf. (**C**) A significant number of *ccndx* morphant embryos exhibited shorter primary motor axons as compared to *ccndx* morphants (panels a and b), and co-injection of mouse *ccnd1*, *ccnd2*, or *ccnd3* mRNA was unable to rescue this defect (panels c, d, and e). Panel f: graph showing the proportion of embryos with signals lower than those in control MO-injected embryos at 24 hpf.

At the time of writing, cyclin Dx has been identified only in zebrafish and *Xenopus*; mammals and chicken appear to express cyclin D3 instead. To explore the functional relationships between members of the mammalian cyclin D family, we injected cyclin Dx morphants with cyclin D1, D2, or D3 mRNA. The *olig2* and *isl1* signals were lost from the motor neurons of *ccndx* morphants (Fig. 6A, panels b and h). However, the expression of *isl1* and *olig2* could not be restored by overexpression of mouse *cyclin D1*, *D2*, or *D3* mRNA (Fig. 6A, panels c, d, e, f, h, i, j, k and l). In addition, overexpression of any one of these genes was not able to significantly restore GFP-labeled primary motoneurons of Tg:(isl1:GFP) transgenic line (Fig. 6B) and the length of primary motor axons in *zccndx* morphants (Fig. 6C). These data suggest that mouse cyclin D1, D2, and D3 do not have similar functions to that of zebrafish and *Xenopus ccndx*.

Taken together, these results indicate that zebrafish cyclin Dx is specifically required for the formation of motor neuron progenitors during development. Its expression is regulated by HIF2a through binding to two HRE sites in the promoter region. However, we discovered that mouse cyclin D1, D2, and D3 do not share the role of zebrafish cyclin Dx in maintaining the pool of motor neuron progenitors, and subsequent promotion of *isl1* and *olig2* expression and primary motor axon development.

## Discussion

During the early stages of ventral neural tube development, three types of cells are generated: floor plate cells, motor neurons, and interneurons. The differentiation of these cells is initially triggered by the notochord, and later by floor plate cells through the Sonic hedgehog (Shh) signaling pathway^49^. Five progenitor domains in the ventral neural tube, consisting of four interneurons (V0 to V3) and one motor neuron (pMN), are defined by Shh signaling. In the present study, the zebrafish *cyclin Dx* transcript was observed to be localized to pMN, distal from the midline and partially overlapping with expression of *oligo2* and *isl1* (Fig. 1). Moreover, the *zccndx* gene was observed to be essential and required for the formation of motor neuron progenitors, and later, expression of the *olig2* and *isl1* genes (Figs 2 and 3), through a process regulated by HIF2α (Figs 4 and 5). Mammals lack the *ccndx* gene (it either evolved after the divergence of mammals and other chordates or was lost from the mammalian lineage), but the mammalian cyclin D genes, *ccnd1*, *d2*, and *d3*, were unable to restore the expansion of motor neuron progenitor pools in mutants, or rescue subsequent expression of *isl1* and *olig2* in *ccndx* morphants (Fig. 6).

The *cyclin Dx* gene was first cloned and characterized in *Xenopus*^21^. In this study, we cloned a *cyclin Dx* ortholog from zebrafish. Although the amino acid sequence identity between zebrafish and *Xenopus cyclin Dx* is only 36%, the similar expression patterns of these genes in the motor neurons of hindbrain and spinal cord, and their specific function in the maintenance of pMNs, confirm that zebrafish *cyclin Dx* is a homolog of *Xenopus cyclin Dx*. Data mining revealed that zebrafish cyclin Dx has higher identities with the predicted sequences of *cyclin Dx* from other bony fish, such as medaka (59%), tetraodon (57%), and fugu (60%) (Supplementary Fig. 3). These genes were placed in the same group upon phylogenetic tree analysis using MEGA software^50^. However, no homolog of this gene is present in the genome of human or mouse. Therefore, such a *cyclin Dx* family may be present only in lower vertebrates and *Xenopus*, and not in mammals or chicken. Instead, mammals and chicken possess the *cyclin D3* gene. However, based on the importance of cyclin Dx in maintaining the pool of motor neuron progenitor cells, it is possible that a cyclin Dx-like protein may be encoded by the mammalian genome, but has not yet been identified due to the great evolutionary variation between fish and mammals.

Oxygen concentration is implicated in stem cell self-renewal and differentiation^51^. During hypoxia, increased expression of hypoxic response factors, such as HIF2α, regulate several cellular processes and signal transduction^52^. The HIF2*α* has been reported to be stabilized by association with ARNT to form a hetero-dimer leading to activate downstream target genes^53^. In zebrafish, there are two Arnt1 homologues, Arnt1a and Arnt1b^54^. While HIF2α has been demonstrated to be required for the differentiation of neural progenitor cells in the hindbrain and spinal cord through its downstream effects on the Birc5a and Birc5b genes, by forming a hetero-dimer with ARNT1A^32^. In this study, we have demonstrated that *ccndx* is one of the downstream target genes of HIF2α/ARNT1A, and such regulation may be responsible for the formation of motor neuron progenitor cells, but not interneurons, during zebrafish early development. Importantly, the expression of the *ccndx* gene is essential for the expression of both *isl1* and *olig2* genes for primary neuron development. The regulation of *ccndx* gene expression is thus critical for primary motor neuron development and motor neuron progenitor cell renewal. In summary, our data indicate that zebrafish cyclin Dx plays a key role in the proliferation and specification of motor neuron progenitor cells.

## Additional Information

**How to cite this article**: Lien, H.-W. *et al.* Zebrafish cyclin Dx is required for development of motor neuron progenitors, and its expression is regulated by hypoxia-inducible factor 2α. *Sci. Rep.* **6**, 28297; doi: 10.1038/srep28297 (2016).

## Supplementary Material

Supplementary Information
