# Supplementary material for: Zebrafish cyclin Dx is required for development of motor neuron progenitors, and its expression is regulated by hypoxia-inducible factor 2α
Source: Sci Rep. 2016 Jun 21;6:28297. doi: 10.1038/srep28297 (PMC4915019; doi:10.1038/srep28297)
Supplement: Supplementary Information [file srep28297-s1.pdf]

Zebrafish cyclin Dx is required for the development of motor neuron progenitors, and its expression is regulated by hypoxia-inducible factor 2α

Huang-Wei Lien[1], Rey-Yue Yuan[2], Chih-Ming Chou[3], Yi-Chung Chen[1], Chin-Chun Hung[4], Chin-Hwa Hu[5], Sheng-Ping L. Hwang[1], Pung-Pung Hwang[1], Chia-Ning Shen[6], Chih-Lung Chen[6], Chia-Hsiung Cheng[3,*], and Chang-Jen Huang[4,*]

[1]Institute of Cellular and Organismic Biology, Academia Sinica, Taipei 115, Taiwan;
[2]Department of Neurology, School of Medicine, College of Medicine, Taipei Medical University, Taipei 110, Taiwan;
[3]Department of Biochemistry and Molecular Cell Biology, School of Medicine, College of Medicine, Taipei Medical University, Taipei 110, Taiwan;
[4]Institute of Biological Chemistry, and [6]Genomics Research Center, Academia Sinica, Taipei 115, Taiwan;
[5]Department of Bioscience and Biotechnology, National Taiwan Ocean University, Keelung, Taiwan

*To whom correspondence should be addressed:
Dr. Chang-Jen Huang
Institute of Biological Chemistry, Academia Sinica, 128, Sec 2, Academia Rd., Taipei 115, Taiwan.
Tel: 886-2-2785-5696; Fax: 886-2-2788-9759
E-mail: cjibc@gate.sinica.edu.tw
Dr. Chia-Hsiung Cheng
Department of Biochemistry, School of Medicine, College of Medicine, Taipei Medical University, 250, Wu-Hsing St., Taipei 110, Taiwan.
Tel: 886-2-2736-1661c ext. 3156; Fax: 886-2-2735-6689
E-mail: chcheng@tmu.edu.tw

Keywords: Zebrafish; cyclin Dx/ccndx; cyclin D3/ccnd3; hypoxia-inducible factor 2α/HIF2a; islet1/isl1; oligo2/olig2; motor neuron progenitor

RB binding domain

```
zccnDx   : MSVSLWCEEDSGRDRTHQASVRAPWDPSASGQ-RVIQRLLQSEERYLPSSLYISLVQREPQ--RREELAK :  67
xccnDx   : MEGRLLCCESV-------TELRARADPVLMQS-RVLMKLLALEERYIPSASYFRCVQRDIQPYMRHMLTC :  62
hccnD1   : MEHQLLCCEVE-------TIRRAYPDANLLND-RVLRAMLKAEETCAPSVSYFKCVQKEVLPSMRKIVAT :  62
xccnD1   : --MELLCCEVD-------TIRRAHLDRNLITD-RVLQTMLKAEETCCPNVSYFKCVQKEILPHMRKIVAT :  60
zccnD1   : MEHQLFCCEVD-------TIRRAYQDSNLLND-RVLQTMLKAEENYLPSPNYFKCVQKEIVPKMRKIVAT :  62
hccnD2   : --MELLCHEVD-------PVRRAVRDRNLLRDDRVLQNLLTIEERYLPQCSYFKCVQKDIQPYMRRMVAT :  61
xccnD2   : --MELLCCEGD-------TVRRAQPDPALLLDDRVLHNLLTVEERYLPQCSYFKCVQKDIQPFMRRMVAT :  61
zccnD2A  : --MELLCLEMD-------TIIRARPDPNLLYDDRVLQSLLTIEERFLPQCSYFKCVQKDIQPFMRRMVAT :  61
zccnD2B  : --MELFCLEKD-------TVVRAQKDPNIFCDERVLQSLLTVEDRYVPQGPYFKCVQKDIQPFMRKMVAT :  61
hccnD3   : --MELLCCEGTR------HAPRAGPDPRLLGDQRVLQSLLRLEERYVPRASYFQCVQREIKPHMRKMLAY :  62
```

Cyclin Box

```
zccnDx   : WTMEVCCECDCDESVFPLAVSLLDRYLSATLSLPVSPSCLAAACILLASKVTESDTVSADTLCAAAEYDF : 137
xccnDx   : WMLEVCEDQKCGEEVFPLAVNCLDRYLSLVPVEKRHLQLLGATCLFLASKLRESKPMTAESLCMYSDHCF : 132
hccnD1   : WMLEVCEEQKCEEEVFPLAMNYLDRFLSLEPVKKSRLQLLGATCMFVASKMKETIPLTAEKLCIYTDNSI : 132
xccnD1   : WMLEVCEEQKCEEEVFPLAMNYLDRFLSVKTLRKSQLQLLGATCMFLASKMKETIPLTAEKLCIYTDNSI : 130
zccnD1   : WMLEVCEEQKCEEEVFPLAMNYLDRFLSVEPTKKTRLQLLGATCMFLASKMKETVPLTAEKLCIYTDNSV : 132
hccnD2   : WMLEVCEEQKCEEEVFPLAMNYLDRFLAGVPTPKSHLQLLGAVCMFLASKLKETSPLTAEKLCIYTDNSI : 131
xccnD2   : WMLEVCEEQRCEEEVFPMAMNYLDRFLAVIPTRKCHLQLLGAVCMFLASKLKETIPLTAEKLCIYTDNSI : 131
zccnD2A  : WMLEVCEEQKCEEEVFPLAMNYLDRFLAVVPTRKCNLQLLGAVCMFLASKLKETRPLTAEKLCIYTDNSI : 131
zccnD2B  : WMLEVCEEEKCEDDVFPLAMNYLDRFLAAVPTRKCYLQLLGAVCLFLASKLKACQPLSARKLCMYTDNSI : 131
hccnD3   : WMLEVCEEQRCEEEVFPLAMNYLDRYLSCVPTRKAQLQLLGAVCMLLASKLRETTPLTIEKLCIYTDHAV : 132
```

Repressor Domain

```
zccnDx   : LSANLREMERVVLATLRWDVLAVTPQDFIPLFLRTLGELRDGDGHTGDFLTTMRRHGDTLVAMCVCDSRF : 207
xccnDx   : TDKELLAMELLVLNKLKWDLEVVTPREYLPHFLELLNIPAEK-------RPQVRKHSETFIALCTTDCTF : 195
hccnD1   : RPEELLQMELLLVNKLKWNLAAMTPHDFIEHFLSKMPEAEEN-------KQIIRKHAQTFVALCATDVKF : 195
xccnD1   : RPEELLLMELLILNKLKWDLASVTPHDFIEHFLNKMPLTEDT-------KQIIRKHAQTFVALCATDIKF : 193
zccnD1   : RPGELLQMELLALNKLKWDLASVTPHDFIEHFLAKLPIHQSS-------KQILRKHAQTFVALCATDVNF : 195
hccnD2   : KPQELLEWELVVLGKLKWNLAAVTPHDFIEHILRKLPQQREK-------LSLIRKHAQTFIALCATDFKF : 194
xccnD2   : KPQELLEWELVVLGKLKWNLAAVTPHDFIEHILRKLPLPKDK-------LLLIRKHAQTFIALCATDFNF : 194
zccnD2A  : RPQELLEWELVVLGKLKWNLAAVTPNDFIEHIMRKLPLPEDK-------LELIRKHVQTFIALCATDFNF : 194
zccnD2B  : TSQQLL-WELVVLSKLKWNLAAITPLDFIEHILHKLPFHEDR-------LTLIRKHTQTFIALCATDHSF : 193
hccnD3   : SPRQLRDWEVLVLGKLKWDLAAVIAHDFLAFILHRLSLPRDR-------QALVKKHAQTFLALCATDYTF : 195
```

```
zccnDx   : LGTPPSLVAAAALNSALRGLRARSAGEMS---LMTAALATLCQTDVALLQCCTELIDGALRERLRTG--- : 271
xccnDx   : IALPPSMVAAASVAAAVTGLQLQSPGPSYSSLASINLLAHAIHCDPSLLRACQEQIEISLESSVQR---- : 261
hccnD1   : ISNPPSMVAAGSVVAAVQGLNLRSPNNFLSYYRLTRFLSRVIKCDPDCLRACQEQIEALLESSLR----Q : 261
xccnD1   : ISNPPSMIAAGSVAAAVQGLNLGNADSVFSTQRLTLFLSQVIKCDPDCLRACQEQIESLLESSLR----Q : 259
zccnD1   : IASPPSMIAAGSVAAAVQGLYLKSTDSCLSSQNLTNFLSQVIRSDPDCLRSCQEQIESLLESSLR----Q : 261
hccnD2   : AMYPPSMIATGSVGAAICGLQQDEEVSSLTCDALTELLAKITNTDVDCLKACQEQIEAVLLNSLQ----- : 259
xccnD2   : AMYPPSMIATGSVGAAICGLQLDVGETSLSGDSLTEHLAKITSTDVDCLKACQEQIESVLVSSLR----- : 259
zccnD2A  : AMYPPSMIATGSVAAAICGLQLNSTNHSLWGDNLTELLAKITNTEVDVLKACQEQIERVLMNNLREGRRQ : 264
zccnD2B  : TMYPPSMIATGCVGAAVCGLQSSQSNQSLWGDNLMELLAKITNTELDCLKSCQEQIEQLLTDSLKES--Q : 261
hccnD3   : AMYPPSMIATGSIGAAVQGLG----ACSMSGDELTELLAGITGTEVDCLRACQEQIEAALRESLREA--- : 258
```

```
zccnDx   : AQEQKD--------GDIEEEERASTPTDLREIDF : 297     Identity(Similarity)
xccnDx   : AQRNR-----VSESKSVDEPERSSTPTDVQDIDL : 290     36%(54%)
hccnD1   : AQQNMDPKAAEEEEEEEEEVDLACTPTDVRDVDI : 295     32%(51%)
xccnD1   : AQQQHN--TSSDTKNMVEEADISCTPTDVRDVNI : 291     32%(51%)
zccnD1   : AQQHIS----TETKRVEEDVDLSCTPTDVRDINI : 291     33%(52%)
hccnD2   : QYRQD--QRDG--SKSEDELDQASTPTDVRDIDL : 289     34%(51%)
xccnD2   : QTRQQTQQRNS--SKSVDELDQASTPTDVQDINL : 291     33%(51%)
zccnD2A  : QQKQQQQEQGGQRSKALDDQDQSSTPTDVRDINL : 298     33%(52%)
zccnD2B  : QQHQQQQQDGRASNKGTQSQNLSCTPTDVRDVNL : 295     31%(50%)
hccnD3   : SQTSSSPAPKAPRGSSSQGPSQTSTPTDVTAIHL : 292     34%(50%)
```

Supplementary Figure. 1

The *zccndx* gene encodes a D-type cyclin. Sequence alignment of cyclin D proteins from zebrafish, *Xenopus*, and human. The predicted RB binding sites, Cyclin Boxes, and Repressor Domains are indicated. The identities and similarities of *zccndx* with other cyclin D proteins are also shown.

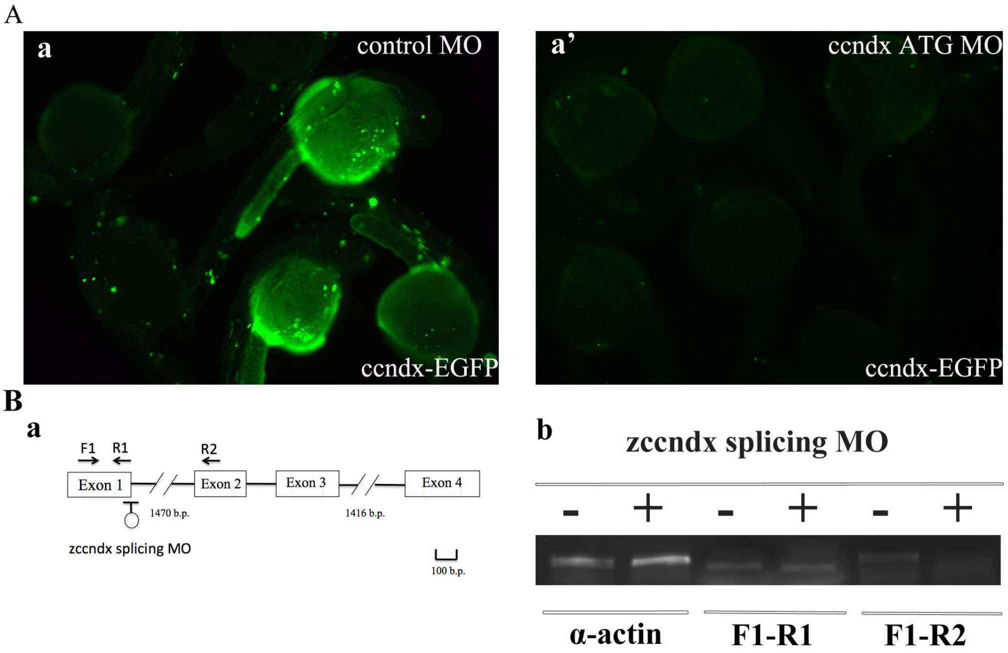

Supplementary Figure. 2

Control experiments for morpholino specificity. The specificities of the MOs against *ccndx* were confirmed using pCMV-GFP reporter plasmids containing the MO target sequence. The pCMV-GFP reporter plasmids bearing the MO target sequence were injected into zebrafish embryos together with the control MO or *ccndx* ATG MO (A, panels a and a'). All images were taken from zebrafish embryos at 48 hpf. (B) Zebrafish embryos were injected with splice-blocking MOs against *ccndx*. The efficiency and specificity of *ccndx* splicing MO were confirmed by RT-PCR with two primer sets (panel a); the RT-PCR results are illustrated in panel b.

```
zCcndx  : MSVSLWCEEDSGRDRTHQASVRAPWDPSASGQRVIQRLLQSEERYLPSSLYISLVQR--EPQRREELAKW :  68
xCcndx  : MEGRLLCCESVTE-------LRARADPVLMQSRVLMKLLALEERYIPSASYFRCVQRDIQPYMRHMLTCW :  63
meCcndx : --QAQGCTGGSPQ-------LRAAWDPTVSGHRVIQRLLHLEERYMPSMLYTTLIQR--DPERREELAKW :  59
TnCcndx : -VPVQYALADTPR-------LRAGWDPSASGQRVIHRLLQVEERYAPSLLYVSLTQQ--EPRRREELAQW :  60
fuCcndx : --MEIICLRCSSQ-------LRAGWDPSVSGCRVIQRLLHVEERYSPSVLYVSLIQQ--EPGRREELTKW :  59

zCcndx  : TMEVCCECDCDESVFPLAVSLLDRYLSATLSLPVS------PSCLAAACILLASKVTESDTVSADTLCAA : 132
xCcndx  : MLEVCEDQKCGEEVFPLAVNCLDRYLSLVPVEKRH------LQLLGATCLFLASKLRESKPMTAESLCMY : 127
meCcndx : ALEVCCDCGCDEAVFPRSVSLMDRFLSASLSLAVS------PYCLAAGCVLIASKLTECDGVTADSLCAA : 123
TnCcndx : ALEVCCDCGCAEAVFPLAVSLMDRFLAGSLAPPVWPVRPLWPVCLAAGCILIASKLTECETVTAERLCAA : 130
fuCcndx : TLQVCCDCGCDEAVFPLAVSLVDRFLSALLTLPVSPVS---PVCLAAGCILIASKLTECETVTAELLCAA : 126

zCcndx  : AEYDFLSANLREMERVVLATLRWDVLAVTPQDFIPLFLRTLGELRDGDGHTGDFLTTMRRHGDTLVAMCV : 202
xCcndx  : SDHCFTDKELLAMELLVLNKLKWDLEVVTPREYLPHFLELLNIP-------AEKRPQVRKHSETFIALCT : 190
meCcndx : AEYSFQPSDLREMERVILSTLRWDTAAVTPQDFLPHFLASVGE--QG-DAEREMLSTLRRHSDTLAAMCA : 190
TnCcndx : AQHHFQPSNLRDMERVILSTLRWDTAAVTPQDFLPHFLASLEE--GGGTWEPDLLSMLRRHSDTLASMCA : 198
fuCcndx : AEHDFLPSSLRDMERLILATLRWDTAAVTPQDFLPHFLASLEE--RGGTFGTELLSTLRRHSDTLASMCV : 194

zCcndx  : CDSRFLGTPPSLVAAAALNSALRGLRAR--SAGEMSLMTA-ALATLCQTDVALLQCCTELIDGALRERLR : 269
xCcndx  : TDCTFIALPPSMVAAASVAAAVTGLQLQSPGPSYSSLASINLLAHAIHCDPSLLRACQEQIEISLESSVQ : 260
meCcndx : CDSRFLGAPPSLVAAASLNCALRGLSNK--GPTQLSKSSE-ALAELCQTDLVVLQCYSEMIESALRQRLR : 257
TnCcndx : CDSRFLGTPPSLVAAASLNCALRGLGRA--GPAELALLGE-VLAALCRTDVAVLQCCSEMIEGALRQRLR : 265
fuCcndx : CDSQFLGTPPSLVAAASLNCALRGLGRT--CHTELVFLGE-ILAALCQTDVAVLQCCSEMIEGALRQRLR : 261

zCcndx  : TGAQE---QKDGDIEEEERASTPTDLREIDF : 297      Identity    Similarity
xCcndx  : R-AQRNRVSESKSVDEPERSSTPTDVQDIDL : 290        36%          55%
meCcndx : GGLQQPPMEKDEEMENE-RPGTPTDMREINF : 287        59%          76%
TnCcndx : RGLERGASEKEEEVENE-RPGTPTDMRDIDF : 295        57%          74%
fuCcndx : SGLQRGPSEKGEEVESE-RPGTPTDMRDIDF : 291        60%          76%
```

Supplementary Figure. 3

Amino acid sequence alignments of zebrafish, *Xenopus*, medaka, tetraodon, and fugu ccndx proteins. Alignments were generated using CLUSTAL X. Identical amino acids are shown in white on a black background.
